# Insulin Resistance, Type 1 and Type 2 Diabetes, and Related Complications 2017

**DOI:** 10.1155/2017/1478294

**Published:** 2017-11-15

**Authors:** Joseph Fomusi Ndisang, Alfredo Vannacci, Sharad Rastogi

**Affiliations:** ^1^Department of Physiology, University of Saskatchewan College of Medicine, 107 Wiggins Road, Saskatoon, SK, Canada S7N 5E5; ^2^Department of Neurosciences, Psychology, Drug Research and Child Health Section of Pharmacology and Toxicology, University of Florence, Viale Pieraccini 6, 50139 Florence, Italy; ^3^The Medical Affairs Company, Cardiovascular, Troy, MI 48085, USA

The global escalation of obesity and diabetes in developed and developing nations poses a great health challenge. Obesity is one of the major causes of type 2 diabetes. Type 1 diabetes is primarily due to the autoimmune-mediated destruction of pancreatic beta cell leading to insulin deficiency [1–3]. This is usually accompanied by alterations in lipid metabolism, enhanced hyperglycemia-mediated oxidative stress, endothelial cell dysfunction, and apoptosis [1–3]. Similarly, in type 2 diabetes, increased glucotoxicity, lipotoxicity, endoplasmic reticulum-induced stress, and apoptosis lead to the progressive loss of beta cells [1–5]. While type 1 diabetes is characterized by the presence of beta cell autoantibodies, a combination of peripheral insulin resistance and dysfunctional insulin secretion by pancreatic beta cells is implicated in the pathogenesis of type 2 diabetes [1–3]. However, both forms of diabetes are associated by a wide variety of complications such as cardiomyopathy, nephropathy, and neuropathy. Although insulin resistance has traditionally been associated with type 2 diabetes, mounting evidence indicates that the incidence of insulin resistance in type 1 diabetes is increasing [6]; therefore, novel mechanistic approaches deciphering insulin resistance are needed. Many pathophysiological factors are implicated in insulin resistance [5, 7]. Although the exact natures of these factors are not completely understood, it is widely accepted that oxidative stress, inflammation, and genetic, habitual, environmental, and other epigenetic factors play a significant role.

In the past two decades, significant strides have been made in elucidating important mechanisms associated with insulin resistance, overt diabetes, and related cardiometabolic diseases. However, more intense research is still needed for a more comprehensive understanding of the pathophysiological profile of insulin resistance in diabetes, especially in situations where diabetes is comorbid with other chronic diseases. Therefore, this special issue is a collection of research and review papers that address a broad range of mechanisms associated with insulin resistance, type 1 diabetes, type 2 diabetes, and related cardiometabolic complications. A common pathophysiological destructive force in type 1 and type 2 diabetes is the high levels of advanced glycation end products generated by hyperglycaemia [1–3]. To unveil further insights on advanced glycation end products, T. Okura et al. wrote an article on the putative pathophysiological role of advanced glycation end products on deregulation of insulin signalling in type2 diabetes. To further expatiate on dysfunctional insulin signalling, F. De C. Cartolano et al. investigated the impact of insulin resistance on lipid metabolism at preclinical level and found that insulin resistance and diabetes are powerful predictors of quantitative and qualitative features of lipoprotein dysfunction and are directly associated with increased atherogenic risk.

Dyslipidaemia, obesity, and visceral adiposity are common risk factors for insulin resistance, type 2 diabetes, and cardiovascular complications [8, 9]. To shed more insight on this theme, C. Fujii et al. investigated the relationships between the composition of free fatty acids and metabolic parameters and found that serum linoleic acid is negatively correlated with the accumulation of visceral fat and insulin resistance. On the other hand, Y. Long et al. investigated the effects of overexpressing gamma-glutamyltransferase on insulin sensitivity and found the short-term overexpression of liver-specific gamma-glutamyltransferase ameliorates insulin sensitivity. Although liver disease commonly occurs in diabetes, other organ complications including cardiomyopathy, neuropathy, and nephropathy are highly documented [1, 2, 4, 5]. To underscore the role of the kidney in diabetes and hypertension, U. Kampmann et al. gave their insights on effects of renal denervation on insulin sensitivity in nondiabetic patients with treatment for resistant hypertension in an article featuring in this special issue. On the other hand, to give further insight on diabetic neuropathy, L. Luo et al. wrote an article underscoring gene profiles of neurotrophin-mitogen-activated protein kinase (MAPK) signalling in patients with diabetic peripheral neuropathy.

Besides obesity, another important habitual factor that affects the development of insulin resistance and type2 diabetes is sedentary lifestyle. Moreover, sedentary lifestyle is one of the modifiable risk factors of type 2 diabetes and the value of exercise to improve insulin signalling and glucose metabolism cannot be overemphasized [10]. Accordingly, in a research article, C. J. Gaffney et al. investigated how acute restoration of normoglycemia affected energy metabolism during exercise in nonobese patients with type 2 diabetes. The authors reported that insulin-induced normoglycemia increased blood glucose during subsequent exercise without altering overall substrate utilization. Similarly, in another article on exercise, S. Benedini et al. underscored the benefits of sports in enhancing insulin sensitivity and glucose metabolism and suggested a putative role of the exercise-induced myokine and irisin in the beneficial effects of exercise on glucose metabolism. Irisin is a protein produced in the muscles during exercise [11]. Generally, small increments of irisin levels in the blood trigger a parallel increment in energy expenditure in mammals with no changes in movement or food intake [11].

Besides exercise, weight-loss surgeries are referred to as bariatric surgery is adopted to reduce health risks associated with obesity [12]. Among the different types of bariatric surgeries is Roux-en-Y gastric bypass surgery [12]. In a related article featuring in this special issue, X. Zhao et al. explored the impact of Roux-en-Y gastric bypass surgery on cardiovascular risk in diabetic patient comorbid with obesity. The authors showed that the Roux-en-Y gastric bypass surgery is an effective strategy to mitigate cardiovascular risk in diabetic patients with comorbid of obesity. In addition to surgery, another emerging therapeutic pathway featuring in this special issue is a review article by J. A. David et al. where the authors highlighted the role of the nuclear-factor-E2-related-factor-2 (Nrf2)/kelchlike ECH-associated-protein-1 (Keap1)/antioxidant response element (ARE) pathway as a target for prevention, prognosis, and treatment of type 2 diabetes. The Nrf2/Keap1/ARE has been well documented for its cytoprotective effects, with potential therapeutic applications [13].

Collectively, the articles featuring in this special issue cover a wide breadth of topics of great interest and would benefit a wide audience.

*Joseph Fomusi Ndisang* *Alfredo Vannacci* *Sharad Rastogi*
